# TLR3 Plays Significant Roles against HBV-Associated HCC

**DOI:** 10.1155/2015/572171

**Published:** 2015-04-23

**Authors:** Xiao-lan Chen, Yu-yin Xu, Li Chen, Gui-lan Wang, Yin Shen

**Affiliations:** ^1^Department of Nephrology, Affiliated Hospital of Nantong University, Nantong, Jiangsu 226001, China; ^2^Department of Pathological Anatomy, Nantong University, Nantong, Jiangsu 226001, China

## Abstract

Toll-like receptor 3 (TLR3) is a pattern-recognizing receptor that is involved in immune signaling and plays a crucial role in survival by being able to recognize various viral components including double-stranded RNA (dsRNA). The role of TLR3 in hepatocellular carcinoma (HCC) with hepatitis B virus (HBV) infections is not well understood. To investigate the ability of TLR3 in regulating HBV replication in HCC, 80 cases of human HCC were collected and their tissue microarray was made. In HCC cells, the expression and location of TLR3, hepatitis-associated virus, and interstitial immunoreactive cells were assayed with immunohistochemical staining. The apoptosis of tumor cells was also detected by TUNEL stain. Correlations between TLR3 expression and HBV infection, interstitial immunoreactive cells, and cells apoptosis in HCC were investigated. In addition, we explored whether TLR3 agonist dsRNA can inhibit HepG2.2.15 cells secreting HBV. We found that the cytoplasmic expression of TLR3 in HCC is positively related to HBsAg infection and HCC with cirrhosis and promotes interstitial immunoreactive cells infiltration and cancer cells apoptosis. In HepG2.2.15 cells, dsRNA inhibited the secretion of HBV and induced apoptosis. These results indicate that TLR3 signaling activity may be involved in immune responses against HBV in HCC.

## 1. Introduction

Hepatocellular carcinoma (HCC) is one of the most prevalent malignant tumors and a leading cause of cancer-related deaths globally [1, 2]. In recent studies conducted in Asia and Northern America, the estimated risk of developing HCC was observed to increase by 25–37-fold in hepatitis B surface antigen (HBsAg) carriers compared with noninfected patients [3, 4]. HBV frequently causes liver inflammation, hepatic damage, and subsequent cirrhosis. The development of liver cirrhosis is recognized as a major step in HCC pathogenesis because it occurs in 80%–90% of HCC [5]. To further investigate the clinical features of HBV-infected HCC and develop more effective therapeutic strategies, considerable efforts have recently been exerted in exploring the molecular mechanisms involved in the development and progression of HBV-associated HCC. Previous studies demonstrated that T cells, NK cells, and antigen-presenting cells (APC) inhibit HBV replication when they are activated by alpha-galactosylceramide, interleukin-12 (IL-12), IL-18, and an agonistic anti-CD40 antibody injection, respectively [6, 7]. Collectively, these results suggest that HBV replication can be controlled by innate immune response if it is activated in the liver.

TLR3 recognizes double-stranded RNA (dsRNA), messenger RNA (mRNA), and the synthetic ligand polyinosinic: polycytidylic acid [poly(IC)] [8, 9] and TLR3 is unique among TLRs in the fact that it does not signal through MyD88, but rather, it uses a distinct adaptor protein, TRIF (TIR domain-containing adaptor-inducing IFN-*β*). TLR3 signaling may induce two downstream pathways, the inflammatory or the apoptotic pathway. The inflammatory pathway is mediated mainly by Rip1 and leads to NF-*κ*B activation. The apoptotic pathway, on the other hand, was shown to be mediated by Rip3 and results in caspase-8 activation [10, 11]. It has been evidenced that the TLR3 detects intracellular viral dsRNA and subsequently activates NF-*κ*B via the TRIF pathway [12]. Previous studies reported that TLR3-induced IFN response was enhanced in hepatocytes isolated from patients with HCV infection. This hyperresponsiveness could be mimicked in native PHHs consistently stimulated with low dose of poly I:C. The data suggested that durable activation of TLR3 by low doses of viral replicative intermediates increases the sensitivity to viral invasion [13]. These findings shed new light on the relevance of TLR3 in the pathogenesis of HBV-infected HCC. In this regard, exogenous activation of TLR3 represents an attractive therapeutic strategy to combat chronic viral pathogens such as HBV and HCV.

In the present study, we detected the expression of TLR3 in human HBV-associated HCC tissues and its relation to HBV infection. Furthermore, we stimulated HepG2.2.15 cells by dsRNA to determine the relative contributions of TLR3 to HBV replication and the possible antiviral mechanism of activating TLR3 in HBV-associated HCC.

## 2. Materials and Methods

### 2.1. Patient Material

The study group included 68 males and 12 females who were enrolled from the Third People's Hospital of Nantong and Haimen People's Hospital between January 2011 and December 2013, ranging in age from 34 to 76 years, with a mean age of 51.5 years. The patients were selected according to the following criteria: (a) having primary HCC and (b) being previously untreated and with surgery as the first treatment. Therefore, analysis of the data in this series would reflect actual impact of the tumor biology on the clinical outcome. All patients were diagnosed and histopathologically confirmed with HCC and had complete clinical and pathological records including medical records, chest roentgenograms, whole body computed tomography films, and bone and brain scanning data. The surgery records were reviewed and the confirmed pathological diagnosis, tumor size, related hepatitis/liver cirrhosis, metastasis, and serum alpha fetoprotein (AFP) values, and other relevant data were analyzed. Negative controls were established from matched adjacent nontumor liver tissues (ANT) which were cut from the area 2–5 cm away from HCC nodules. They were derived from 80 cases of HCC. The study was approved by the Ethics Committee of Third People's Hospital of Nantong and Haimen People's Hospital, and all the patients signed informed consent.

### 2.2. Tissue Microarrays

Tissue microarrays (TMA) were constructed according to the method of E. Qun (Patent number ZL 2008 1 0022 170.4). Briefly, all HCC tissues were stained by H&E and reviewed by two histopathologists. Representative areas free from necrotic and hemorrhagic materials were marked in paraffin blocks. Two cylindrical tissue cores (1.6 mm diameter) were removed from the donor blocks and transferred to the recipient paraffin blocks, and their planar array positions were noted. Three different TMA blocks were constructed. Each contained over 100 cylinders and the final TMAs consisted of 80 cases of HCC and 80 cases of ANT. Consecutive sections (4 *μ*m thick) were cut from the array blocks and placed on adhesion microscope slides for immunohistochemical staining.

### 2.3. Immunohistochemistry Staining

The Envision+/DAB analysis method was performed on formalin-fixed, paraffin-embedded 4 *μ*m sections from all patients for the detection of TLR3, HBsAg, and HBcAg in cancer cells. Ten consecutive TMA sections were prepared from TMA block and stained. The paraffin slides were dewaxed in xylene. For antigen retrieval, slides were heated at 95°C for 10 min in sodium citrate buffer (10 mM sodium citrate monohydrate, pH 6.0) in microwave. The slides were allowed to cool for 20 min at room temperature and then incubated in Envision + peroxidase blocking solution (Dakocytomation, Glostrup, Denmark) for 5 min and rinsed with 0.05% Tris-buffered saline (TBS)/Tween 20 buffer, pH 7.4. The slides were then incubated with primary antibodies for 30 min at room temperature. Rabbit anti-TLR3 monoclonal antibodies (diluted 1 : 100) were obtained from Abcam. Rabbit monoclonal antibodies against human HBsAg and HBcAg (diluted 1 : 100) and mouse monoclonal antibodies CD3, CD68, CD56, and CD117 (diluted 1 : 100) were all purchased from Fuzhou Maixin Biotech. Co., Ltd., China. The slides were washed with 0.05% Tween 20 in TBS (pH 7.4). Detection was achieved with the DAKO Envision+/HRP system (DAKO, Carpinteria, CA, USA). The color was developed by a 15 min incubation with a diaminobenzidine (DAB) solution (DAB kit IL1-9032) (Fuzhou Maixin Biotech. Co., Ltd., China), and sections were slightly counterstained with hematoxylin. Positive controls and negative controls (TBS was substituted for primary antibody at the same concentration) were performed for each immunohistochemical run.

TLR3 located on cytomembrane and cytoplasm of HCC cells. HBsAg and and HBcAg located on cytoplasm and nucleus of HCC cells. CD3, CD68, CD56, and CD117 located on cytoplasm of interstitial immunoreactive cells, each separately representing T cells, Kupffer cells, NK cells, and mast cells.

### 2.4. Terminal Deoxynucleotidyl Transferase-Mediated dUTP Nick End Labeling (TUNEL) Staining

TUNEL detection kit (Promega, USA) was employed for the detection of neuronal apoptosis. In brief, paraffin-embedded sections were deparaffinized and dehydrated. After washing in phosphate-buffered saline (PBS), sections were treated with 20 *μ*g/mL proteinase K for 20 min. After washing in PBS thrice (3 min for each), sections were rinsed with 0.3% Triton X-100 for 10 min followed by washing in PBS. These sections were incubated with TUNEL reaction mixture at 37°C for 1 h. Following washing in PBS thrice (3 min for each), sections were treated with HRP conjugated streptavidin (1 : 200, Beijing Zhongshan Biotech. Co., Ltd.) at 37°C for 30 min. After washing in PBS thrice (3 min for each), sections were treated with 0.04% DAB and 0.03% H_2_O_2_ at room temperature for visualization for 8–12 min. After washing in water, counterstaining was done with hematoxylin followed by mounting with resin. In the negative control, TUNEL reaction mixture was replaced with PBS. The positive control sections were pretreated with DNase I for 10 min followed by TUNEL staining. Cells with blue granules in the nucleus were regarded as positive for TUNEL. We counted the TUNEL-positive cells in the RGC layer of each sample in 10 HPF (400x). Positivity was graded according to the percentage of tumor cells stained as negative (0–5%), weak (6–30%), and strong (30–100%).

### 2.5. dsRNA Synthesized

dsRNA was designed based on cell surface TLR3 sensitive viral sequences in human echovirus, human poliovirus, enterovirus 70, and coxsackievirus from GenBank. Furthermore, the viral sequences were submitted for basic local alignment search tool (BLAST) analysis (http://www.ncbi.nlm.nih.gov/blast/) to ensure that the sequence was not homologous to human genes. The target sequence of dsRNA was CCGGCCCCUGAAUGCGGCUAAUC (23 nt) [14] and was synthesized by Biomics Biotechnologies Co., Ltd., Jiangsu, China.

### 2.6. Cell Culture

The human HCC cell line HepG2.2.15 with secreting HBV was purchased from Ruijin Hospital (Shanghai, China). Cells were maintained in Dulbecco's modified Eagle's medium (DMEM) (Gibco BRL, Grand Island, NY, USA) supplemented with 20% fetal bovine serum (FBS), 2 mM L-glutamine, and 100 U/mL penicillin-streptomycin mixture (Gibco BRL) at 37°C and 5% CO_2_ in a humidified chamber.

### 2.7. qRT-PCR

HepG2.2.15 cells were seeded into the wells of a 6-well culture plate and allowed to grow until 80% confluence. Subsequently, these cells were treated with the dsRNA (10 *μ*g/mL) and PBS (negative control), respectively. After treatment at 37°C for 24 hours, total RNA was isolated from HepG2.2.15 cells using TRIzol (Invitrogen, Carlsbad, CA). qRT-PCR was performed for TLR3, HBsAg, and HBcAg using an ABI 7700 Sequence Detection System (Applied Biosystems). Cycling conditions for amplification were 95°C for 3 min; 35 cycles at 95°C for 45 sec, 60°C for 45 sec, and 72°C for 30 sec; and, finally, 72°C for 7 min. The primers are listed in Table 1. Each human gene expression was normalized to GAPDH mRNA copies from the same sample.

### 2.8. Western Blot

HepG2.2.15 cells were treated as described by qRT-PCR analysis. Immunoprecipitation cell lysis buffer was added to the wells, and the plate was put on ice for 30 minutes; then cells treated as described above were scraped, and cell lysate was removed to 1.5 mL EP tubes and spun for 15 minutes. The supernatant was taken for the experiment. Protein concentrations were determined by an optical density (Eppendor, Hamburg, Germany). Proteins were separated by 10% polyacrylamide gel electrophoresis and then transferred onto polyvinylidene fluoride (PVDF) membranes (Millipore, USA) at 350 mA for 2 h, which was later soaked for 2 h on a blocking solution (Tris-buffered saline containing 5% nonfat dry milk and 0.01% vol/vol Tween 20). Membranes were incubated for 18 h at 4°C with anti-TLR3 monoclonal antibodies (diluted 1 : 1000, Abcam) and anti-human HBsAg and HBcAg (diluted 1 : 500) antibodies (Fuzhou Maixin Biotech. Co., Ltd., China). Anti-*β*-actin mouse monoclonal antibody (Sigma, USA) was used as internal control. After incubation, the membrane was washed 3 times, and peroxidase-conjugated secondary antibodies (ICN Laboratories, Irvine, CA; diluted 1 : 10,000) were added and incubated for an additional one hour. Reaction was visualized by the ECL chemiluminescence detection system (Pierce, USA) on radiographic films (Koda, USA) on BIO-RAD ChemiDoc XRS (Bio-Rad, USA). The results were analyzed using ImageJ software.

### 2.9. Flow Cytometry Assay

Flow cytometry (Beckman Coulter, Fullerton, CA, USA) was used to determine the apoptotic rate. The HepG2.2.15 cells treated with dsRNA or PBS were suspended in a 500 *μ*L binding buffer (Becton Dickinson, USA), incubated with 5 *μ*L Annexin V-FITC/PI (Becton Dickinson, USA) and 5 *μ*L propidium iodide (PI) (Becton Dickinson, USA) for 15 minutes. Phosphatidyl serine translocation to the cell surface serves as an indicator of early apoptotic cells; therefore, Annexin V-positive and PI-negative cells were identified as apoptotic cells. The apoptotic rate was determined using Cell Quest software (FCM, Becton Dickinson).

### 2.10. Statistical Analysis

Statistical analysis was performed using SPSS 17.0 for Windows. Differences between groups were evaluated with *χ* ^2^, Fisher exact test, and Spearman rank correlation analysis. A *P* value <0.05 was considered significant.

## 3. Results

### 3.1. TLR3 Expression and Location in HCC and ANT Tissues

**Figure 1:**
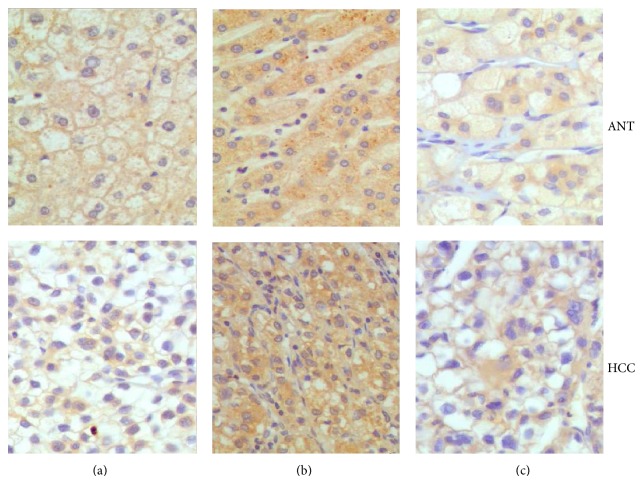
TLR3 expression and location in HCC and ANT tissues. TLR3 exhibited cytoplasm (a), cytomembrane staining (b), and cytoplasm/cytomembrane (c), respectively, in HCC and ANT. (IHC magnification ×200).

The expressions of TLR3 in HCC and ANT were examined by immunohistochemical analysis, which were showed in cytoplasm, cytomembrane, or cytoplasm/cytomembrane. No significant difference was observed in positive rate and expression pattern of TLR3 between HCC and ANT samples (*χ* ^2^ = 1.7309, *P* = 0.189, *χ* ^2^ = 5.512, and *P* = 0.064) (Figure 1 and Table 2).

### 3.2. Association of TLR3 Expression with Clinicopathologic Features of HCC

**Figure 2:**
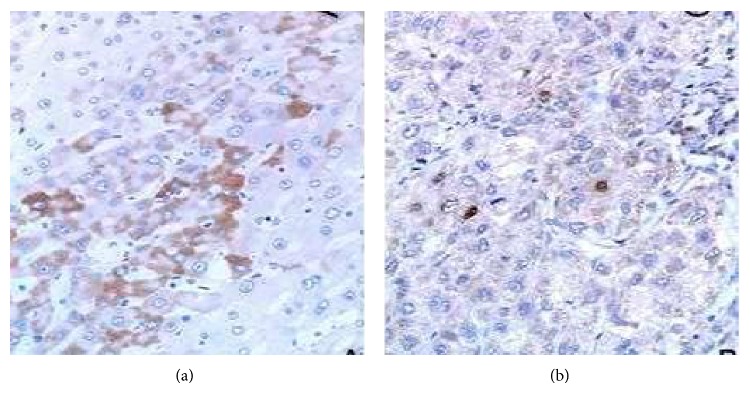
HBsAg and HBcAg expression in HCC tissues. (a) HBsAg expression in the cytoplasm; (b) HBcAg expression in the cell nucleus. IHC stain, magnification ×200.

In this paper, the ratio of HCC tissues with HBsAg and HBcAg infection was 27.5% (22/80) and 15% (12/80), respectively (Figure 2). The correlation of TLR3 expression with the clinicopathologic characteristics of HCC was investigated (Table 3). Intratumoral TLR3 positive rate was negatively related to the serum AFP levels (*χ* ^2^ = 6.2411, *P* = 0.012) and positively related to HBsAg infection (*χ* ^2^ = 9.5477, *P* = 0.002) and tumor with cirrhosis background (*χ* ^2^ = 26.5278, *P* = 0.000). And yet TLR3 expressing pattern was related to HBsAg infection (*χ* ^2^ = 12.999, *P* = 0.002). However there were no correlations between TLR3 positive rate and age, gender, HCC size, grates, and HBcAg infection (*P* > 0.05).

### 3.3. Association of TLR3 Expression with Interstitial Immunoreactive Cells

**Figure 3:**
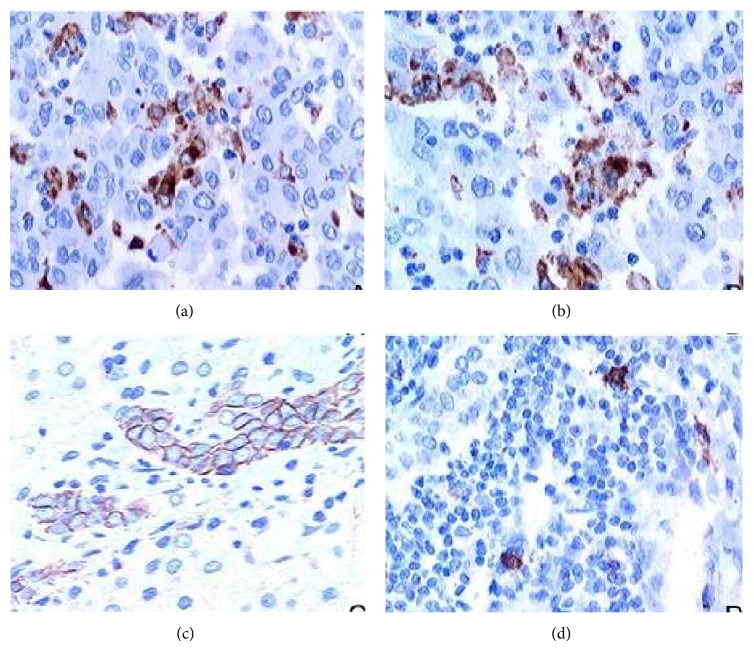
Interstitial immunoreactive cells infiltration, T cells (a), Kupffer cells (b), NK cells (c), and mast cells (d) were, respectively, marked CD3, CD68, CD56, and CD117 antibodies by immunohistochemical staining in HCC (IHC ×400).

Interstitial immunoreactive cells, T cells, Kupffer cells, NK cells, and mast cells were, respectively, marked CD3, CD68, CD56, and CD117 antibodies by immunohistochemical staining (Figure 3). TLR3 expression was positively correlated with interstitial infiltration of T cell (*χ* ^2^ = 10.944, *P* = 0.001 < 0.05; *r* = 0.370, *P* = 0.001 < 0.05), Kupffer cells (*χ* ^2^ = 7.230, *P* = 0.007 < 0.05; *r* = 0.301, *P* = 0.007 < 0.05), and NK cells (*χ* ^2^ = 5.792, *P* = 0.014 < 0.05; *r* = 0.269, *P* = 0.016 < 0.05). TLR3 membrane expression related to interstitial infiltration of T cells (*χ* ^2^ = 6.084, *P* = 0.017 < 0.05; *r* = 0.276, *P* = 0.013 < 0.05) and NK cells (*χ* ^2^ = 3.855, *P* = 0.071 > 0.05; *r* = 0.220, *P* = 0.050) and TLR3 cytoplasm expression related to Kupffer cells infiltration (*χ* ^2^ = 8.727, *P* = 0.003 < 0.05; *r* = 0.330, *P* = 0.003 < 0.05). But TLR3 positive rate and expressing patterns have no correlation with mast cells (*P* > 0.05) (Table 4).

### 3.4. Association of TLR3 Expression with Tumor Apoptosis in HCC

**Figure 4:**
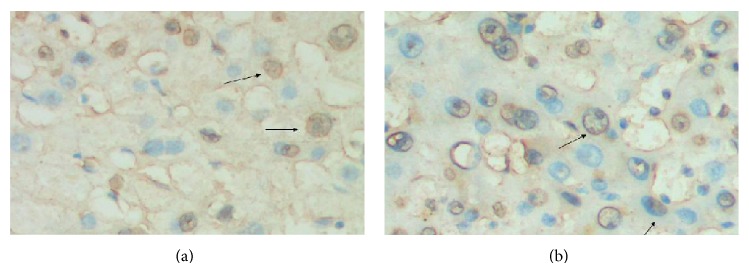
TUNEL detected apoptosis in HCC tissues. Apoptotic nuclei were stained in brownish yellow (indicated by arrow), while normal nuclei were stained in blue (magnification ×400).

The results of TUNEL detection showed that the apoptosis index in HCC tissues was 55% (44/80) (Figure 4). The expression of TLR3 positively correlated with HCC apoptosis (*χ* ^2^ = 11.2517, *P* < 0.001; *r* = 0.3354, *P* < 0.001). No significant difference was observed between HCC apoptosis and TLR3 expression patterns in HCC (*P* > 0.05) (Table 5).

### 3.5. Stimulation of TLR3 Inhibits HBV Secretion of HepG2.2.15 Cells

**Figure 5:**
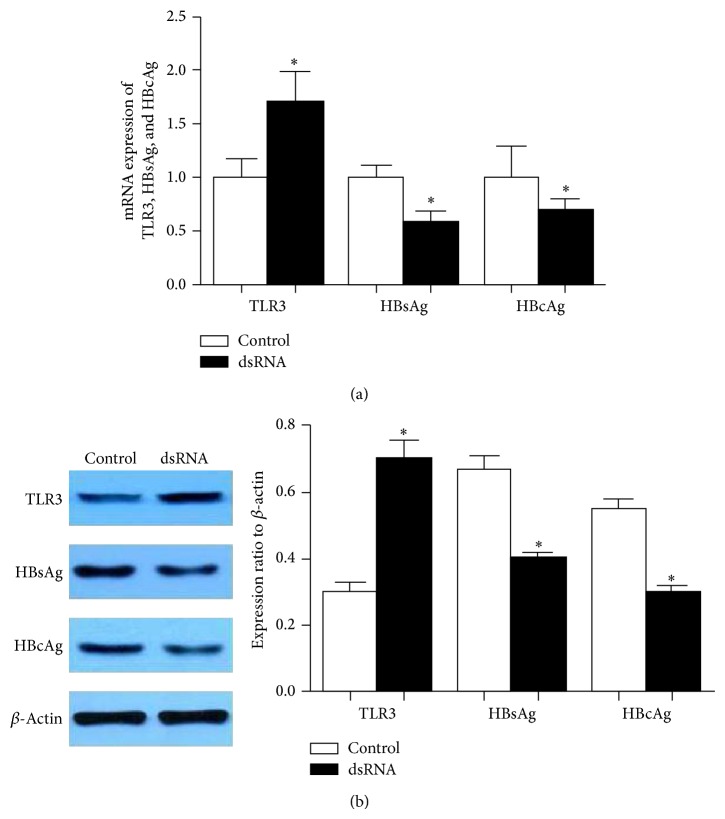
dsRNA stimulated the expression of TLR3 and inhibited the expression of HBsAg and HBcAg in HepG2.2.15 cells. (a) By qRT-PCR. (b) By western blotting (^∗^ *P* < 0.05 versus control group).

TLR3 is generally believed to play an important role in the innate immune response against viral infection, including viral hepatitis infection, although controversial results have been reported [15]. The controversial reports on the role of TLR3 in the antiviral defense may be due to the difference in the type of viruses, the type of cells that are infected, the viral load, its model of infection (endoplasmic versus cytoplasmic), and stage of infection. We tested the expression of HBsAg and HBcAg of HepG2.2.15 cells by using qRT-PCR and western blot analysis after treatment with dsRNA. The results showed that when the cells were treated with dsRNA, the levels of HBsAg and HBcAg were greatly reduced (Figures 5(a) and 5(b)). These results showed that HBsAg and HBcAg protein were less expressed in dsRNA treated HepG2.2.15 cells.

### 3.6. dsRNA Inducing Cell Apoptosis of HepG2.2.15 Cells

**Figure 6:**
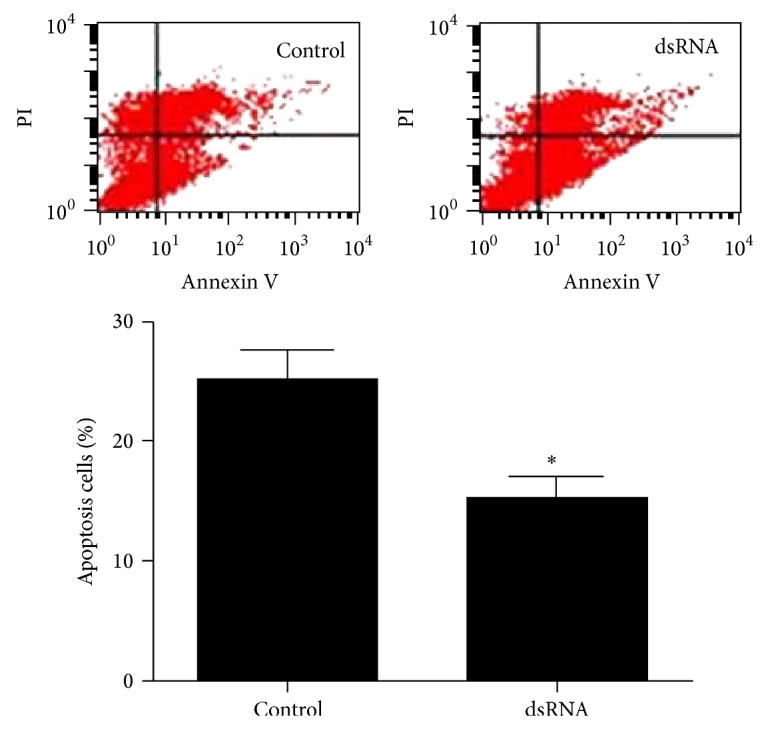
Flow cytometry detection of apoptosis. dsRNA induced HepG2.2.15 cell apoptosis (^∗^ *P* < 0.05 versus control group).

Inhibition of cell growth could result from the induction of apoptosis, cell growth arrest, and/or the inhibition of growth. We investigated whether activation of the expression of TLR3 induced apoptosis in HepG2.215 cells. The Annexin V-FITC/PI double staining was used as indicator of apoptosis. Activation of TLR3 increased the percentage of Annexin V-positive/PI-negative cells (Figure 6). We found that cells treated with dsRNA for 24 h resulted in decreased cell viability and increased cell apoptosis.

## 4. Discussion

HBV as the main prevalent infectious agent plays important roles in inducing severe liver diseases. Previous studies demonstrated that, during prolonged forms of HBV infection including chronic, asymptomatic, and occult forms, patients are unable to eradicate HBV from hepatocytes completely [16]. Several lines of evidences suggested that a synergistic interaction between environmental carcinogens and HBV-carcinogens may play a critical role in the carcinogenesis of HCC [17, 18]. The main mechanisms responsible for development of the forms of hepatitis B are yet to be identified.

It has been evidenced that TLRs are essential for the recognition of invading pathogens and serve as an important link between innate and adaptive immunity. TLR3 is an endosomal receptor for dsRNA and is expressed in several subsets of immune cells, including dendritic cells and natural killer (NK) cells. TLR3 is also expressed by fibroblasts [19], lung epithelial cells [20], hepatocytes [21], and several types of tumor cells. TLR3 is involved in antiviral responses and the production of type I interferons (IFNs) [22]. It is the only TLR that signals exclusively through the MyD88-independent pathway, which activates TRIF and IRF3 and results in production of anti-inflammatory mediators such as IFN-*β*, IL-10, TGF-*β*, and RANTES [23]. Previous studies found that TLR3 is an important modulator of HCC progression and is a potential target for novel immunotherapy [24].

In the present study, we investigated the significance and relationship between TLR3 expression and HBV infection, apoptosis, and interstitial immunoreactive cells infiltration in HCC. We found that TLR3 was generally expressed in HCC tissues (positive rate 58.75%) and ANT (positive rate 68.75%), located in the cytoplasm and cytomembrane of HCC cells. These results are similar to those by Yoneda et al. [25]. In this study, expression level of TLR3 was negatively correlated with serum AFP levels. AFP is a protein that can be expressed by HCC cells, with extremely complicated biologic activities. Studies have shown that AFP plays double roles in both inhibiting the immune system and promoting the growth of cancer cells. These results indicate that the expression level of TLR3 was positively correlated with HBsAg infection and HCC with cirrhosis background, the higher levels and cellular HBsAg infection, the higher positive rate of TLR3. The results also indicate that the HCC with HBV infection may upregulate the synthesis of dsRNA which were involved in replication or transcription process and then activate TLR3, which in turn promote interstitial immunoreactive cells and induce inflammatory cytokine production. The above process activate the body's adaptive immune response that will against viruses. This hypothesis had been confirmed by detecting the relationship between TLR3 expression, HBsAg expression, and cirrhosis background. Consequently, high expression of TLR3 is based on high levels of HBV infection. The results suggest the importance of TLR3 in antiviral immunity in vivo. In addition, we also examined the potential antiviral effect of TLR3 in vitro. dsRNA activation of TLR3, which signals through a TRIF-dependent pathway, induces expression of various protective mediators and anti-inflammatory cytokines, such as poly I:C, a synthetic dsRNA analog, in human astrocytes [26]. Here, we show that dsRNA can inhibit the secretion of HBsAg and HBCAg of HepG2.2.15 cells. Preconditioning with 10 *μ*g/mL dsRNA significantly increased TLR3 expression and decreased HBsAg and HBcAg protein expression, which agrees with the experimental results of Mcclary et al. [27]. The antiviral effects of TLR3 signaling on HCC with HBV infection are likely mediated via stimulating of a variety of cells to produce type I IFN that subsequently inhibits HCV or HBV replication [28–31]. These cells include parenchyma cells in HCC and interstitial immunoreactive cells.

Apoptosis is one of the mechanisms leading to cell death when cells have sustained damage to their DNA or cytoskeleton [32]. In this study, we found that the upregulation of TLR3 can not only anti-HBV but also induce apoptosis of HCC cells. We have shown that the expression of TLR3 has positive correlation with apoptosis by TUNEL staining. The results of experiments in vitro were consistent with it. After dsRNA treatment, HepG2.2.15 cell apoptosis was enhanced and activity was decreased. Zorde-Khvalevsky et al. [33] discovered that, during the initial regenerating phase following partial hepatectomy, TLR3 signaling was induced in hepatocytes, leading to activation of NF-*κ*B and caspase-8 and an increase in Rip3 protein levels. Upon activation, caspase-8 cleaves effector caspases, which leads to cell death by initiating apoptotic program. We reasoned that the TLR3-dependent activation of NF-*κ*B and caspase-8 in hepatocytes could result in an increase in activated IL-1*β*, subsequently inhibiting hepatocyte proliferation and inducing HepG2.2.15 cell apoptosis. In short, the mechanism by which dsRNA activates TLR3 is very complex and further studies will be conducted.

In conclusion, the upregulation of TLR3 plays a crucial role in the process of HBV cleaning and inducing HCC apoptosis in HBV-associated HCC. It suggests that TLR3 activation could represent a powerful and novel therapeutic strategy for the treatment of chronic HBV infection and HBV-associated HCC. However, further studies are required to confirm these findings and to provide better understanding of the TLR3 signaling mechanism in the development of HBV-associated HCC.

## Figures and Tables

**Table 1:** The primers used in the experiments.

Number	Name	Sequence (5′→3′)
Hs-HBsAg	F	TGGTTATCGCTGGATGTGTCTG
	R	CCGTGCTGGTGGTTGAGG

Hs-HBcAg	F	TGTGGTTATCCTGCGTTAATG
	R	GCGTCAGCAAACACTTGG

Hs-TLR3	F	TCCCAGCCTTACAGAGAAGC
	R	CCTGTGAGTTCTTGCCCAAT

Hs-GAPDH	F	GAAGGTGAAGGTCGGAGTC
	R	GAAGATGGTGATGGGATTC

**Table 2:** TLR3 expression in HCC and ANT tissues.

Sample	*N*	Positive *n*		*P*	Expressing pattern^*^			*P*
		+	%		M	P	M/P	
HCC	80	47	58.75	>0.05	8	25	14	>0.05
ANT	80	55	68.75		2	36	17	

^**∗**^P, M, and M/P, respectively, represent the expressing pattern of cytoplasm, cytomembrane, and cytoplasm/cytomembrane.

**Table 3:** Correlation between TLR3 expression and clinicopathologic characteristics in HCC.

Item	*N*	Positive rate		*P*	Expressing pattern			*P*
		+	%		M	P	M/P	
Age (year)								
≤51.5	38	21	55.26	>0.05	4	10	7	>0.05
>51.5	42	26	61.90		4	15	7	
Sex								
Male	68	38	55.88	>0.05	6	20	12	>0.05
Female	12	9	75.00		2	5	2	
AFP (ng/mL)								
<400	64	42	65.63	<0.05	6	22	14	>0.05
≥400	16	5	31.25		2	3	0	
Tumor size								
<2 cm	6	4	66.67	>0.05	1	2	1	>0.05
2–5 cm	49	25	51.02		6	14	5	
>5 cm	25	18	72.00		1	9	8	
Grade								
G1	8	6	75.00	>0.05	0	6	0	>0.05
G2	33	21	63.64		4	10	7	
G3	39	20	51.28					
HBsAg								
+	22	19	86.36	<0.05	2	16	1	<0.05
−	58	28	48.28		6	9	13	
HBcAg								
+	12	7	58.33	>0.05	1	3	3	>0.05
−	68	40	58.82		7	22	11	
Cirrhosis								
+	42	36	85.71	<0.05	5	20	11	>0.05
−	38	11	28.95		3	5	3	

**Table 4:** Association of TLR3 expression with interstitial immunoreactive cells.

TUNEL	*N*	TLR3		*P*	Expressing pattern			*P*
		+	%		M	P	M/P	
CD3								
≥50/HPF	37	29	78.38	<0.05	7	13	9	<0.05
<50/HPF	43	18	41.86		1	12	5	
CD68								
≥5/HPF	48	34	70.83	<0.05	5	21	8	<0.05
<5/HPF	32	13	40.63		3	4	6	
CD56								
≥5/HPF	18	15	83.33	<0.05	4	7	4	<0.05
<5/HPF	62	32	51.61		4	18	10	
CD117								
≥10/HPF	25	15	60.00	>0.05	2	7	6	>0.05
<10/HPF	55	32	58.18		6	18	8	

**Table 5:** Correlation between TUNEL expression and TLR3 expression in HCC.

TUNEL	*N*	TLR3		*P*	Expressing pattern			*P*
		−	+		M	P	M/P	
−	36	27	9	*<*0.05	2	4	3	>0.05
+	44	6	38		6	21	11	
